# Risk Factors for Tuberculosis in Foreign-Born People (FBP) in Italy: A Systematic Review and Meta-Analysis

**DOI:** 10.1371/journal.pone.0094728

**Published:** 2014-04-14

**Authors:** Loredana Ingrosso, Fenicia Vescio, Massimo Giuliani, Giovanni Battista Migliori, Lanfranco Fattorini, Santino Severoni, Giovanni Rezza

**Affiliations:** 1 Department of Infectious, Parasitic and Immune-mediated Diseases, Istituto Superiore di Sanità, Roma, Italy; 2 WHO Collaborating Centre for TB and Lung Diseases, Fondazione S. Maugeri, Care and Research Institute, Tradate, Italy; 3 Migration and Health, WHO European office for investment for health and development, Castello, Venice, Italy; St. Petersburg Pasteur Institute, Russian Federation

## Abstract

In Italy, TB notifications in foreign-born people (FBP) are steadily increasing. To investigate this issue we did a meta-analysis on risk factors for FBP people. A systematic search was performed in PubMed and EMBASE from Jan-1980 to Jan-2013. We analysed HIV status, previous TB-treatment, intravenous drug use and alcohol abuse, and multidrug resistant TB. Odd ratio was used as a measure of effect. One and two-stages approaches were used. In the main analysis we used a 2-stages approach to include studies with only aggregate estimates. Among 1996 references, 18 fulfilled inclusion criteria. In TB-affected FBP people positive HIV-status was about 3 times higher than among Italians, after 1996 when combined antiretroviral therapy for HIV was introduced (OR: 2.91; 95%CI: 1.37; 6.17). No association was found between FBP and intravenous drug users in adults; after 1-stage meta-analysis foreign born people from highly endemic countries had a 4 times higher risk to be multidrug resistant TB than Italian people. Finally, TB-affected FBP were less likely than Italians to be alcoholics (OR: 0.10 95%CI: 0.01; 0.84) or of having received previous TB-treatment (OR: 0.55; 95%CI: 0.43; 0.71). An association of multidrug resistant TB with immigrant status as well as an association of Tuberculosis with HIV-positive status in foreign-born people are major findings of this analysis. Drugs and alcohol abuse do not appear to be risk factors for TB in FBP, however they cannot be discharged since may depend on cultural traditions and their role may change in the future along with the migratory waves. An effective control of TB risk factors among migrants is crucial to obtain the goal of TB eradication.

## Introduction

After more than 100 years from R. Koch's first description of its causative agent *Mycobacterium tuberculosis*, tuberculosis (TB) is still considered a worldwide health problem: in 2011 it was responsible for an estimated 8.7 million new cases, globally[1].

Rapid detection and effective cure of infectious TB patients has represented the main pillar of the national TB programme for decades, with the aim of reducing transmission of *Mycobacterium tuberculosis* within the community[2]. However, an additional strategy aimed at diagnosing and treating latent TB infection in order to sterilize the “reservoir” and prevent future TB cases to occur was conceptualized as TB elimination (defined as <1 sputum smear positive cases per 1 million inhabitants[2]–[4]). This approach is the basis of the new World Health Organization post-2015 strategy[5].

Indeed, in Europe, despite the downward trend observed over the last 20 years, the decrease in TB notification has levelled off: this is possibly due to the slowing of the decline in several European member states and to an increase of notifications in others; furthermore, a general increase of TB notifications has been observed in foreign born people (FBP) living in low endemic countries. This is also true for Italy[1].

The vast movement of people across Europe has steadily changed the epidemiological features of the majority of EU western countries. In Italy, along the decade 1998–2008, the number of TB cases in foreign-born persons has more than doubled and they represent a percentage close to 50% of total cases. In general, although the incidence has decreased in recent years, the immigrant population still has a relative risk of suffering from tuberculosis 10–15 times higher than the Italian population[6], [7]. Almost two-thirds of the cases of tuberculosis in foreigners in 2008 occurred in northern Italy, where immigration is more represented than in other areas of the country and the most affected age groups were those of young adults[6], [7].

The massive influx of people from high endemic regions often living in precarious condition and unable or unwilling, for cultural reasons, to comply with the therapeutic protocols contributes to a scenario in which TB affected people are younger, poorer and more difficult to be timely diagnosed and effectively treated than it was in the past. Furthermore, an increasing number of multi-drug resistant-TB (MDR-TB) strains (i.e. strain resistant to at least isoniazid and rifampicin) are isolated in FBP: this aspect further adds to the complex picture of TB epidemiology in Italy. This relatively new and potentially troublesome epidemiological picture has to be taken into consideration in order to assess the measures required to speed up the downward trend of TB in Italian born people and to reverse the TB escalation observed in FBP, under the vision of improving TB control and eventually reaching TB elimination[8].

Aim of the study was to investigate the role of several factors in relation to TB in FBP; eventually the results of this study may help assess how feasible is the goal of TB elimination in a multi-ethnic and multi-cultural environment. To do so, we performed a meta-analysis of the national scientific literature of the latest 30 years, keeping in mind that we were observing a dynamic process composed by subsequent migration waves changing over the years. We addressed the HIV status, previous TB-treatment (relapsing TB), intravenous drug use (IDU), alcohol abuse and the MDR-TB for their association with Italians or with FBP. When possible, we performed meta-analysis of individual data to better understand the role of HIV positive status and the association between MDR-TB and FBP.

## Materials and Methods

### Ethical standards

Systematic revision and meta-analysis of published data. Informed consent from patients not required.

### Search Strategy, inclusion and exclusion criteria

**Figure 1:**
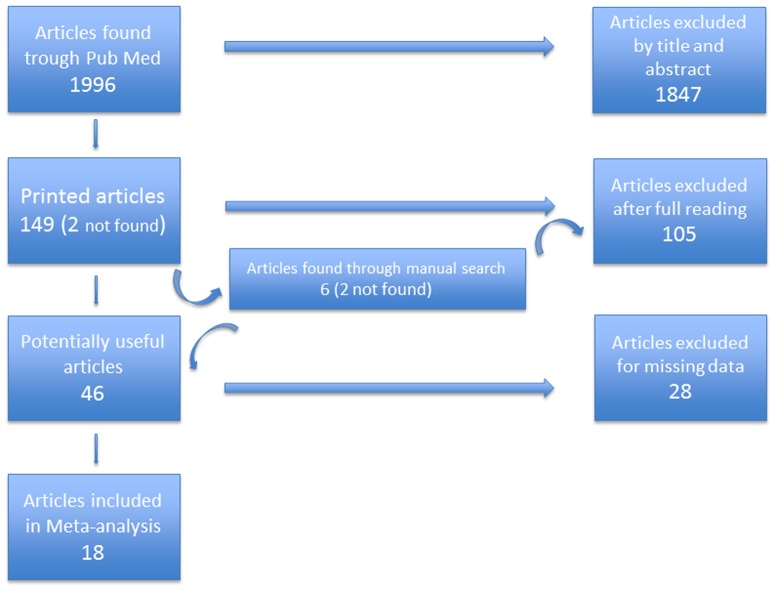
Selection process of studies included in the meta-analysis.

A literature search using the Medline and Embase databases was performed to identify all studies on TB-infection and immigrants published between January 1981 and April 2013. The literature search was undertaken using extended terms for TB and immigration ((“tuberculosis”[MeSH Terms] OR “tuberculosis”[All Fields]) OR TB[All Fields]) AND (“italy”[MeSH Terms] OR “italy”[All Fields]) AND (“1980/01/01”[PDAT]: “2013/01/31”[PDAT]) after a previous attempt with terms like: migrants, immigrants, not born in Italy and foreigner born people showed a great variability in number and variety of articles retrieved (see flow chart, Fig. 1). We therefore decided to maintain the search as large as possible in order to find the vast majority of the relevant articles. The full test of primary articles was assessed against the inclusion criteria.

### Inclusion and exclusion criteria

Inclusion and exclusion criteria were established before reviewing abstracts and articles. Inclusion criteria were: articles written in English, or Italian; studies involving adult immigrants and description of potential risk factors for pulmonary TB in foreign born people. Studies were excluded for the following reasons: case reports or articles presenting less than 10 cases, articles that dealt with a *Mycobacterium* other than TB, or with extra-pulmonary TB only, when they were limited to selected risk groups like occupational risk groups or prisoners, or were outbreaks reports; we also excluded studies involving adolescents or infants because most of the time they were included in outbreak reports in a very limited area, studies evaluating failure or efficacy of therapy, diagnosis and/or diagnostic methods, articles focussed on antibiotic resistance only, and, finally, articles reporting studies performed outside Italy. Among the articles that survived the exclusion criteria we further excluded 23 studies because they did not provide data about risk factors in immigrants [9]–[30] and (apart from age) [31], 1 study[32] was excluded because excluded HIV+ subjects, 1 study because the TB patients were all HIV positive[33], 1 study because included only children[34], 1 study[35] because included only Caucasians and 1 study[36] because included only immigrants. Particular care was taken to identify and exclude possible duplicate studies by confronting authorship, study population by number, year and setting of the study. In addition, some authors provided individual-level data that exclude any possibility for duplicate material.

### Hypotheses and data extraction

We analysed gender, age, poverty, HIV infection, history of intravenous drug use, alcohol use, MDR-TB and relapsing TB in immigrants compared with Italian population. We also summarised the prevalence of TB infection among immigrants. For these purposes, two investigators (L.I. and F.V.) independently extracted the following information from each study: location, design, year in which the study was conducted, selection criteria, total TB cases. When available, the same information was extracted for new and recurrent TB-cases. The number of TB cases in immigrants, HIV prevalence, HIV prevalence in immigrants, mean and/or median age, mean and/or median age in immigrants, proportion of males participants, the number of males participants who were immigrants, proportion of IDUs, the number of IDUs who were immigrants, the number of alcohol addicts, the number of alcohol addicts who were immigrants, the number of TB cases in low SEP (Socio-Economic Position) participants, the number of TB cases in low SEP immigrants, proportion of MDR-TB (multi drug resistant TB) cases, the number of MDR-TB cases occurred in immigrants, delay in diagnosis, length of stay in Italy.

### Statistical analyses

Individual level data was available for the following studies: [37]–[41]. Meta-analyses of the association between TB prevalence and HIV, IUD, alcohol use and MDR-TB were carried out for studies in which this information was available. The odds ratio (OR) was used as a measure of effect. In some studies, ORs were already available whereas in others these were calculated from reported prevalence of TB in different exposure groups. One and two stages approaches were used. In the main analysis we used a 2 stages approach to include studies for which only aggregate estimates were available. These studies were excluded from stratified analyses.

In the 2 stages meta-analysis we carried out logistic regressions separately for each study and then we combined results by random effect (D+L: Der Simonian - Laird) and fixed effect (M-H: inverse variance) meta-analysis [2], [4]. Random effect meta-analysis was used in the presence of heterogeneity. I^2^ statistics were calculated as a measure of the degree of heterogeneity that was not dependent on the number of studies [3], [5]. Sensitivity analyses were used to identify potential sources of heterogeneity between studies. Meta-regressions were carried out to investigate the impact of selected study characteristics on heterogeneity. To evaluate the effect of cART (combined anti-retro viral therapy) on the association between TB and HIV infection, we carried out stratified analyses for studies conducted before (pre cART) and after 1996 (post cART). Because in cross sectional studies the OR tends to inflate the association between two variables when the prevalence is high we run an additional 2 stages meta-analysis using log binomial regressions to estimate the pooled prevalence ratios.

In the 1 stage meta-analysis we combined all individual data in a single dataset and carried out multilevel logistic regressions with random effect at the study level. We conducted sub-group analyses for cases classified as a new or relapsed and for cases aged 30 or less, and for those aged more than 30. We divided participants in Italians and foreign-born subjects and grouped the latter according to the length of their stay in Italy. Hence we carried out separate analyses to compare the HIV status of immigrant TB patients who lived in Italy less than 2 years, between 2 and 5 years and more than 5 years with that of Italian cases. We also classified countries of origin by endemic level for MDR-TB (as defined by WHO) into 4 categories and carried out a sub-group analysis to investigate differences between low and high endemic countries.

The presence of small study effects was visually assessed by Funnel plots and formally tested by the Egger's modified regression test (Harbord test) [42], [43]. All statistical analyses were performed using Stata 10.0 [44].

## Results

### Study characteristics and methodological quality of studies

Overall, 18 articles (totalling to 7817 immigrants) satisfied the inclusion criteria and were included in the meta-analysis. Of these, 10 enrolled more than 700 individuals, 6 between 300 and 700 and 2 less than 250. The main characteristics of the studies included in the meta-analysis are shown in Tables 1 and 2. The median age of the total population varied from 35.0 [45] to 57.3 [46], when taking in account immigrants only, the median age ranged from 29.6 [37] to 34.2 [46], for the Italian population the median age ranged from 41,3 [29] to 64.1 [11]. In most articles criteria used to define low SEP varied for Italians and immigrants (e.g. for Italians it was not possible to distinguish between low SEP and chronic diseases).

**Table 1:** Characteristics of studies included in the meta-analysis.

id#	Author	Year	Type of Study	Multicentric	Reference population	Sampling	Resident pop	Year of data collection	National level	N° of subjects
[37]	Ambrosetti et al.	1999	survey	yes	aAIPO 46 TB- units (hospital/ambulatory)	all consecutive definite cases	all	1995	yes	778
[47]	Codecasa et al.	1999	survey	-	regional hospital based registry (Milan)	all definite cases	not specif	1993-1996	no	2616
[38]	Ambrosetti et al.	1999	survey	yes	aAIPO 46 TB- units (hospital/ambulatory)	all consecutive definite cases	all	1996	yes	838
[40]	Centis et al.	2000	survey	yes	aAIPO 46 TB- units (hospital/ambulatory)	all consecutive definite cases	all	1998	yes	1162
[41]	Centis et al.	2002	survey	yes	aAIPO 46 TB- units (hospital/ambulatory)	all consecutive definite cases	all	1999	yes	906
[48]	Baussano et al.	2008	cohort/retrospective	-	Registry of pulmonary TB (Piedmont)	all definite cases	only res	2001-2005	no	1564
[39]	Ambrosetti et al.	1999	survey	yes	aAIPO 46 TB- units (hospital/ambulatory)	all consecutive definite cases	all	1997	yes	715
[46]	Santori et al.	2005	cross-sectional/retrospective	-	USL 7 hospital based registry (Siena)	all definite cases	all	1994–2003	no	200
[49]	Bonadio et al	2000	survey	no	Pisa hospital (Pisa)	all consecutive definite cases	all	1996–1998	no	88
[50]	Migliori et al.	2002	survey	yes	22 Laboratory network/46 clinical units	all consecutive definite cases	all	1998–1999	yes	810
[45]	Girardi et al.	1996	survey	no	San Camillo/Spallanzani hospital (Rome)	all new cases	all	1990–1992	no	407
[51]	Codecasa et al.	1991	cohort	no	TB unit Milano (Milan)	all definite cases	only res	1985–1989	no	340
[52]	Moro et al.	2002	survey	yes	23 Lab network/12 clin units (Milan)	all consecutive definite cases	only res	1995–1997	no	581
[53]	Fattorini et al.	2012	survey	yes	30 Lab network	all consecutive definite cases	only res	2008–2010	yes	5267
[54]	Pasticci et al.	2012	survey	no	IDC of Perugia University hospital (Perugia)	all consecutive definite cases	all	1971–2010	no	419
[55]	Odone et al.	2011	surveillace data	no	Emilia Romagna Regional TB surv. (Emilia Romagna)	all consecutive definite cases	all	1996–2006	no	5377
[10]	Baussano et al.	2006	cohort	yes	TB not.reg./TB treat.outc. monit.syst/Lab TB reg/HDR (Piedmont)	all consecutive definite cases	all	2001	no	640
[56]	Nutini et al.	1998	survey	yes	Pulmonology Center of the province of Florence/Careggi hospital./Lab units in the province of Florence (Florence)	all consecutive cases	only res	1992–1995	no	433

a AIPO: Italian Association of Hospital Pneumologist.

**Table 2:** Characteristics of studies included in the meta-analysis.

id#	Author	Year	N	FBP	Male	Age	Age FBP	Relapses	Relapses FBP	Resistance	Resistance FBP	HIV	HIV FBP	SEP	SEP FBP	length of stay in Italy
				(%)	(%)	(median)	(median)	(%)	(%)	(%)	(%)	(%)	(%)	(%)	(%)	(months)
[37]	Ambrosetti et al.	1999	778	21	59	46.1	29.6	-	-	4	8	3	5	10	2	31
[47]	Codecasa et al.	1999	2616	23	-	-	30.5	1	0	1	0	18	9	-	-	36
[38]	Ambrosetti et al.	1999	838	26	56	49.4	30.7	-	-	10	11	3	4	9	3	33
[40]	Centis et al.	2000	1162	28	62	-	31.4	-	-	16	19	3	9	10	2	30
[41]	Centis et al.	2002	906	36	61	47.2	31.9	-	-	9	10	1	2	10	4	32
[48]	Baussano et al.	2008	1564	43	62	55.14	32.2	21	0	-	-	-	-	4	0	-
[39]	Ambrosetti et al.	1999	715	24	56	48.9	33.9	-	-	10	9	2	8	11	5	33
[46]	Santori et al.	2005	200	15	85	56.3	34.2	8	0	3	-	5	13	7	27	-
[49]	Bonadio et al.	2000	88	22	40	54.6	-	28	0	-	-	6	11	-	-	-
[50]	Migliori et al.	2002	810	28	-	-	-	14	8	-	-	-	-	-	-	-
[45]	Girardi et al.	1996	407	29	72	35.0	-	21	0	26	29	8	-	-	-	-
[51]	Codecasa et al.	1991	340	11	56	-	-	-	-	-	-	2	3	20	61	43
[52]	Moro et al.	2002	581	30	63	38.0	-	12	0	22	0	21	18	8	0	42
[53]	Fattorini et al.	2012	5267	51	-	-	-					-				
[54]	Pasticci et al.	2012	419	41	-	-	-					12	13			
[55]	Odone et al.	2011	5377	36	-	-	-					2	4			
[10]	Baussano et al.	2006	640	26	-	-	-					5	5			
[56]	Nutini et al.	1998	433	17	-	-	-					-	-			

The five articles reporting on the AIPO study (Italian Association of Hospital Pneumologists) were performed in Italy along several years[37]–[41] and showed a certain heterogeneity mainly concerning the proportion of immigrants enrolled in each study which varied from 21% [37] in 1995 to 36% in 1999 [41]. Another difference relates to the proportion of HIV infection that decreased from 4% (interval: 2–5 [37]) to 1% (interval: 0–1 [39]) among the Italian patients, and increased from 5% (interval: 2–8 [37]) to 9% (interval: 6–12 [40]) in the group of TB foreign-born patients. Studies [41] included also data from the SMIRA (Studio Multicentrico Italiano Resistenze Antitubercolari: Italian Multicentre Study on Resistance to Antituberculosis Drug) group.

### Association between HIV-TB and immigrations

**Figure 2:**
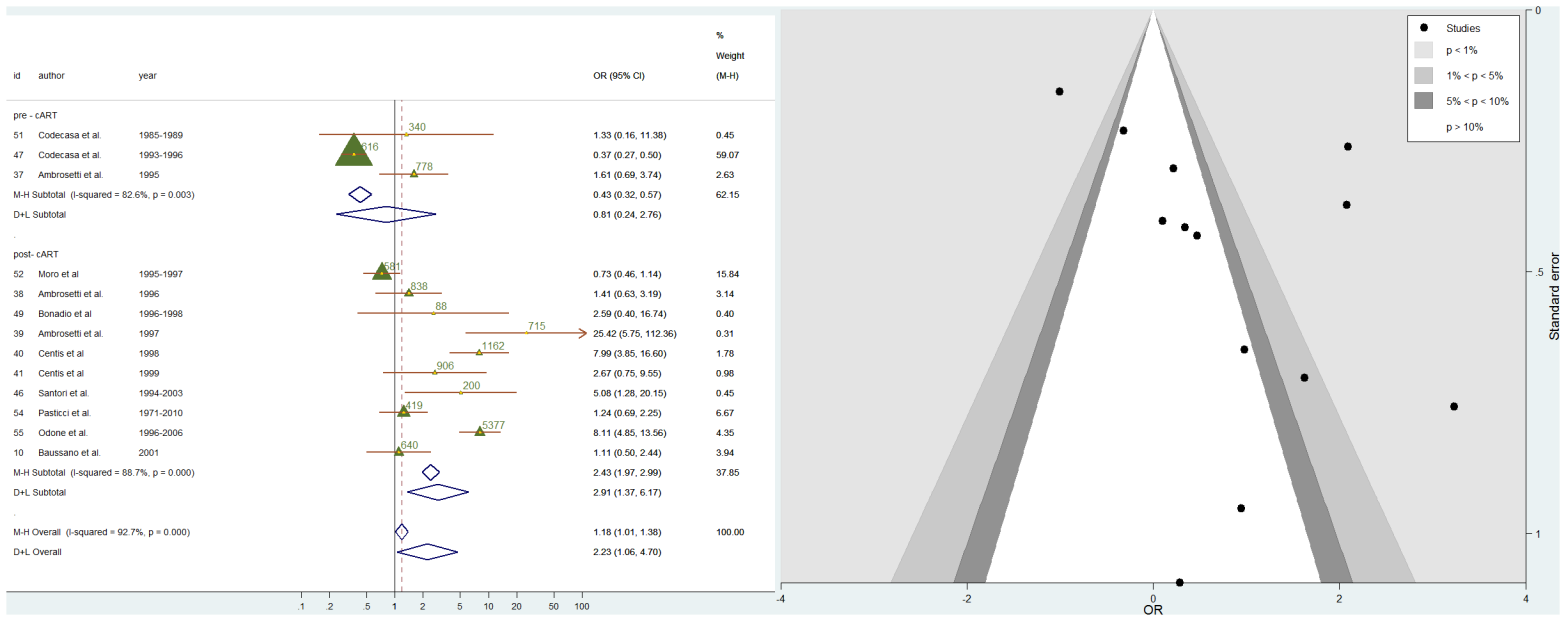
Meta-analysis of the 13 studies included in the 2-stages meta-analysis of the association of HIV positive status with immigration status in TB cases. (a) Forrest plot: the M-H Overall OR was obtained using a fixed effect meta-analysis; the D-L Overall OR was obtained using a random effect meta-analysis. Studies were listed according to year of data collection and grouped for pre-cART (before 1996) and post-cART (after 1996). Triangles are proportional to the study size, (value of study population size is also given). Diamonds represent overall results. Horizontal lines represent 95% confidence intervals. Year, year of data collection. Id, article reference number. 95%CI, 95% confidence intervals. I^2^, statistic which measures the degree of heterogeneity. Weight (M-H), fixed effect weight, based on the sample size of studies. (b) Funnel Plot: Horizontal axis, ORs; Vertical axis, standard error of the log ORs.

Among the studies included in the meta-analysis, 13 took into consideration the relationship between HIV status and TB. Some of them, such as Centis[40], Ambrosetti [39], Santori [46] and Odone[55] reported that immigrant patients affected by TB had a higher probability to be HIV positive than Italian TB patients. On the contrary, Codecasa[47] (fig. 2a) reported that HIV infection was less frequent among immigrants compared to Italians (p<0.01). For the remaining articles [10], [37], [38], [40], [49], [51], [52], [54] the association was weak and not significant. When all the studies were pooled together, there was evidence of association between HIV positive status and immigration (M-H OR: 1.18; 95%CI: 1.01; 1.38; D+L OR: 2.23 (1.06; 4.70). However, both, graphical assessment and tests for heterogeneity showed how the association between HIV and immigration varied between studies (between-study variance 1.14; I^2^: 92.7%). To dissect heterogeneity and investigate the role of combined antiretroviral therapy (cART) in the association TB-HIV we stratified the analyses by year of data collection i.e., before and after 1996. After 1996 we found that the association between TB and HIV infection was strongly represented in immigrants (M-H OR: 2.43 95%CI: 1.97–2.99; D+L OR: 2.91; 95%CI: 1.37; 6.17) whereas this was not the case before 1996 (M-H OR: 0.43 95%CI: 0.32; 0.56; D+L OR: 0.81 95%CI: 0.24; 2.76) (figure 2a). However there was still evidence of heterogeneity (before 1996 between-study variance: 0.68; I^2^: 82.6%; after 1996 between-study variance: 1.07; I^2^: 88.7%). Pooled age-adjusted OR for HIV, estimated by meta-analysis of studies (4 studies carried out after 1996 for which individual level data was available) for which it was possible to obtained age-adjusted estimates was 2.38 (1.49; 3.81). When the immigrants' median age for each study was taken in account in a meta regression analysis, the between-study variance was reduced from 1.14 to 0.92 and I^2^ from 92.7 to 89.8%. When the mean length of stay in Italy as well as immigrants median age was taken into account, the between-study variance and I^2^ were both reduced to 0. The same analysis yielded ORs of 2.54 (1.36; 4.75) for immigrant's median age in years and of 0.70 (0.56; 0.80) for months of stay in Italy. Hence, the length of stay in Italy gives the best explanation for the differences in HIV effect between the studies, since it explains 92.7% of the variation. Individual data one–stage meta analysis stratified by age group, showed that in the older age group, immigrants were more likely to be HIV positive than Italians [OR older than 30 = 3.30 (2.07; 5.26)], whereas the association between HIV and immigration was weak and not statistically significant for the younger subjects [30 years and younger  = 1.46 (0.71–2.99)]. Stratified analyses by length of stay, showed that immigrants who had been in Italy for 2 years or less had the highest OR for HIV [2.60 (1.50; 4.52)]. ORs for those who had been in Italy between 2 and 5 years was still higher than the OR for people born in Italy but lower than the OR for newer immigrants [ORs of 2.19 (1.20; 3.98)]. After 5 years in Italy immigrants and Italian born had similar odds [1.21 (0.47; 3.11)].

### Relevance of small study effect bias

The funnel plot was asymmetric (Fig. 2b). The Harbord method (intercept 3.736;) provided some evidence of small study effect (p = 0.070). We compared results from the fixed and random effects models to investigate the influence of small-study effects. Because there was substantial between-trial heterogeneity, the studies were weighted much more equally in the random-effects analysis than in the fixed-effect analysis. In the fixed-effect analysis Codecasa et al. [47] and Moro et al. [52] obtained 68% and 20% of the weight respectively. Both studies were carried out in Milan where the incidence of TB was higher (14.8 cases×100000) than at the national level (9.1×100000). Milan also had the highest incidence of HIV infection (22×100000) and the highest proportion of foreign-born residents (2.4% of the total resident population) in 1996. Moreover in Milan a large nosocomial outbreak of MDR-TB affected AIDS-hospitalized patients in 1991-1995. In the random-effects analysis the overall estimate was influenced by small studies with large estimates. Most influential studies were Ambrosetti et al.[39] and Centis et al. [40]. Both were high quality surveys where the prevalence of HIV among immigrants was almost double than for the total population. When the analysis was limited to studies carried out after 1996 there was no indication of small study effect (Harbord: intercept  = 1.456, P = 0.578).

### Association between MDR-TB and immigration

**Figure 3:**
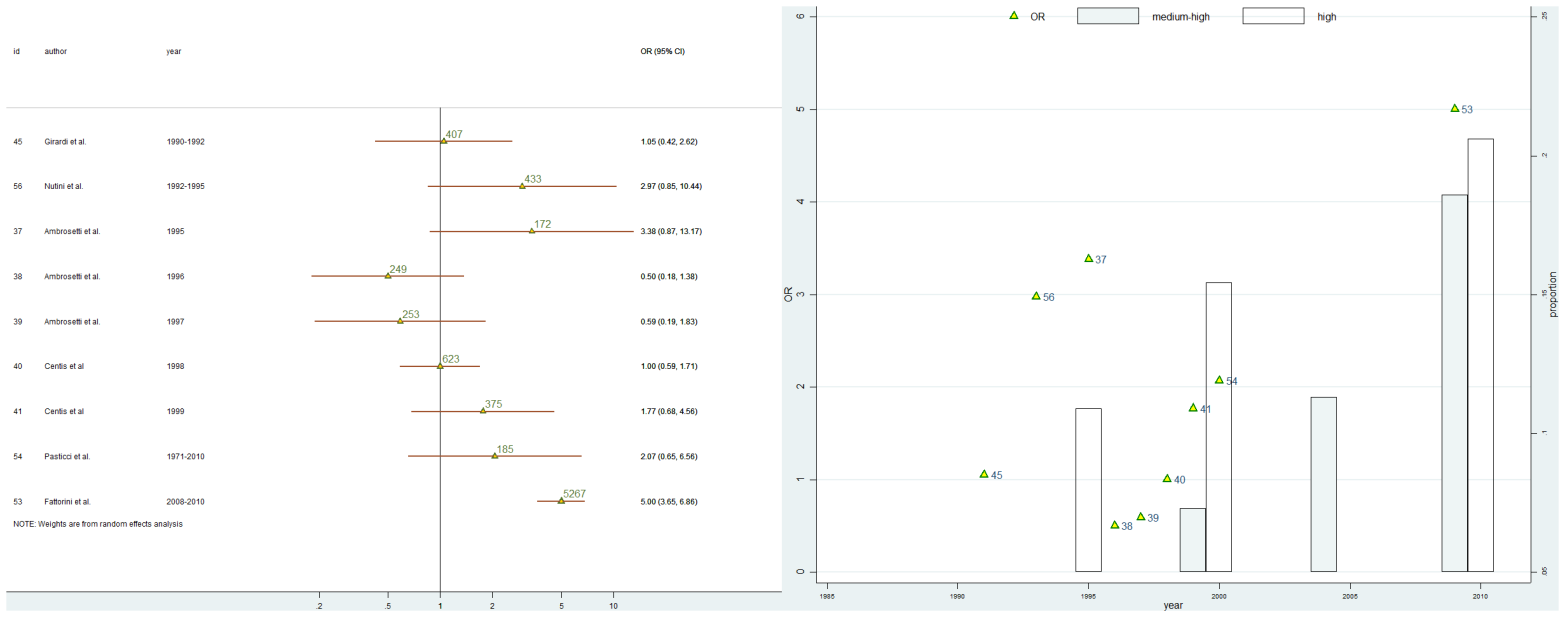
Meta-analysis of the association of MDR-TB and immigration status in TB cases. (a) Forrest plot: triangles are proportional to the study size, (value of population size for each study is also given). Horizontal lines represent 95% confidence intervals. Year, year of data collection. Id, article reference number. I^2^, statistic which measures the degree of heterogeneity. 95%CI, 95% confidence intervals. (b) Scatter plot of the ORs for the association of MDR-TB and FBP (triangle; article id number) from studies included in the meta-analysis per year of data collection. Bar plot of the variation per year of the proportion of immigrants from medium-high (white bar) and high MDR-TB endemic countries (shaded bar) (for countries classification according to their level of MDR-TB endemicity see: Global Tuberculosis Report 2012, WHO 2012[1]). The years-related progressive increment of foreign-born persons from medium to high MDR-TB endemic countries in Italy (sources: ISTAT Tab5 [62]) seems to parallel the increase of ORs of the association of MDR-TB and immigration status in TB cases during the lasts 2 decades.

Nine studies were included in the meta-analysis[37]–[41], [45], [53]–[55]. Apart from Fattorini et al [53] which provided evidence of an association between MDR-TB and immigration status, no association was found by other studies which enrolled a limited number of MDR-TB cases [e.g. the study by Ambrosetti et al. [37] enrolled 9 MDR-TB cases (5 among immigrants - most immigrants were born in high endemic countries for MDR-TB -, and 4 among Italians), the study by Centis et al. [40] enrolled 71 MDR-TB cases (23 immigrants -mostly from countries of low to medium endemic level of MDR-TB- and 48 Italians]. As shown in Fig 3a there was evidence of heterogeneity (84.1% p<0.001).

Articles included in the meta-analysis spanned over 20 years. Because either FBP country of birth or prevalence of MDR-TB may have changed over time (Fig. 3b) we decided not to compute a pooled estimate. An in-depth analysis of all available individual data by random effect multilevel logistic regression (one-stage approach meta-analysis), yielded an OR adjusted for age of 2.40 (1.84; 3.14). Furthermore, classifying countries by their endemic level of MDR-TB (according to WHO criteria) immigrants from medium endemic countries had a OR almost 2 times higher [OR: 1.85 (1.37; 2.50)] than immigrants from low endemic countries and Italians, while those from high endemic countries reached an OR almost 4 times higher than immigrants from low endemic countries and Italians [OR: 3.70 (2.71; 5.04)].

### Association between relapse TB and immigration

**Figure 4:**
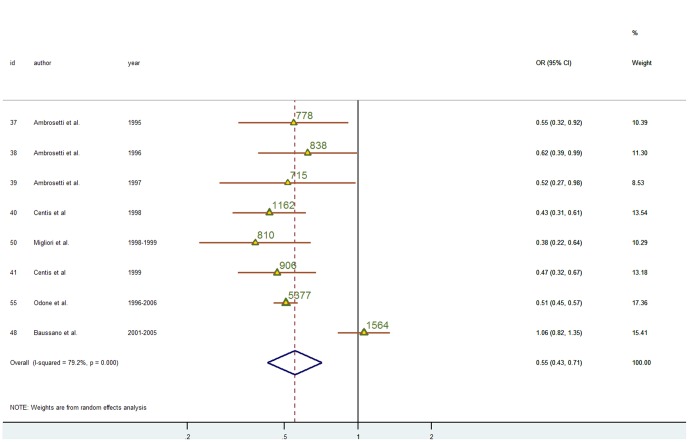
Crude ORs based on 2-stages meta-analysis of the association of relapse-TB and immigration status in TB cases. The Overall OR was obtained using a random effect meta-analysis. Triangles are proportional to the study size, (value of population size for each study is also given). Diamonds represent overall results. Horizontal lines represent 95% confidence intervals. Year, year of data collection. Id, article reference number. I^2^, statistic which measures the degree of heterogeneity. 95%CI, 95% confidence intervals. Id, article reference number. Weight (D-H), random effect weight based on the sample size of studies and the degree of heterogeneity.

Eight studies classified the cases as: new or previously treated (fig. 4). In 7 of them [37]–[41], [50], [55], Italians were more likely than immigrants to have a history of previous treatment. The association was weak and not significant in Baussano[48]. The pooled OR of relapse-TB (see Fig. 4) showed that immigrants were less likely to have a history of previous treatment than Italians (OR: 0.55; 95%CI: 0.43; 0.71). There was evidence of heterogeneity (I^2^ 79.2, p<0.001; tau2 = 0.1044). Taking into account the mean age, the between-study variance and I^2^ were both reduced to 0. In a meta-regression analysis the estimated OR for mean age was 1.10 (1.02; 1.18) and when the analysis was restricted to studies for which individual data were available, the OR adjusted for age was 0.60 (0.47; 0.77). In addition, results from the one–stage meta-analysis stratified by age yielded significant ORs for relapses among older subjects [0.46 (0.35; 0.61)] but not at younger age [OR 1.14(0.71–1.82)].

Since the study by Baussano et al [48] may have introduced bias in our meta-analysis because equate FBP from EU countries (also immigrants from highly endemic Eastern European countries) to Italians, we repeated our analysis after exclusion of this study. The resulting OR did not differ from that obtained from overall analyses (0.59: 0.50; 0.70), but the I^2^ statistic reduced to 0% (p = 0.819) indicating that Baussano et al. [48] differed from the other studies included in the meta-analysis. When the analysis was limited to studies carried out after 1996 the OR was 0.63(0.54; 0.73). The funnel plot was asymmetric but there was no evidence of bias (Harbord: intercept 0.789; p = 0.675).

### Association between IDU and TB for foreigner born subjects

**Figure 5:**
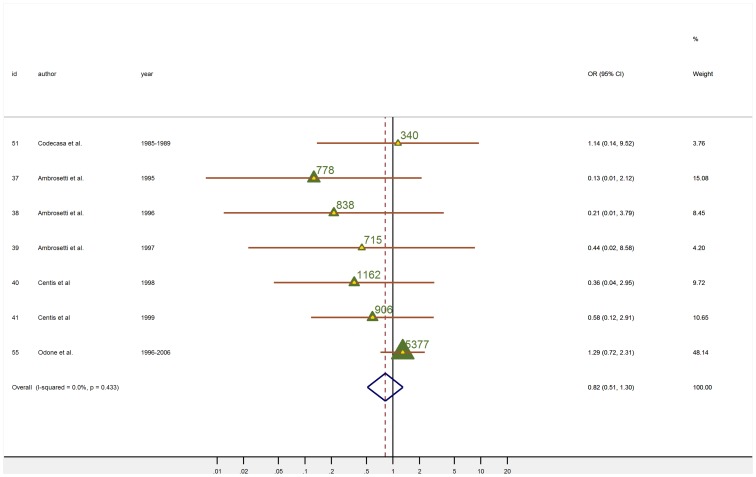
Crude ORs based on 2-stages meta-analysis of the association of IDU and immigration status in TB cases. The overall OR was obtained using a fixed effect meta-analysis (represented by diamond). Triangles are proportional to the amount of information contributed by each study (actual study population numbers are also given). Diamonds represent overall results. Horizontal lines represent 95% confidence intervals. Year, year of data collection. Id, article reference number. I^2^, statistic which measures the degree of heterogeneity. 95%CI, 95% confidence intervals. Id, article reference number. Weight (M-H), fixed effect weight based on the sample size of studies.

Seven studies contributed to this analysis (Fig. 5). The pooled OR was 0.82 (0.51; 1.30). None of the studies was significant *per se*. The funnel plot was asymmetric and there was no evidence of bias (Harbord: intercept: -1.669; p = 0.054). Odone et al. [55] found that TB-affected immigrants aged 20–39 years were less likely to be IDUs than TB-affected Italians. Furthermore, one–stage meta analysis stratified by age group, in articles for which individual data was available, showed that young immigrants were less likely to be ID users than Italians [OR younger than 30 = 0.24 (0.07; 0.90) - OR of 30 and older  = 0.60 (0.21; 1.72)].

### Association between alcohol and TB for foreign-born subjects

**Figure 6:**
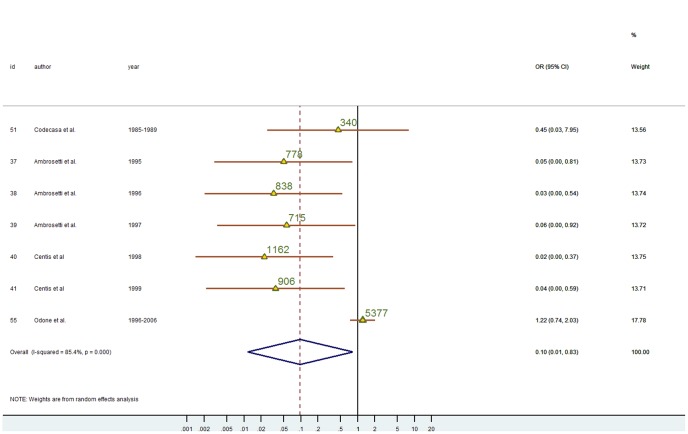
Crude ORs based on 2-stages meta-analysis of the association of alcohol intake and immigration status in TB cases. The Overall OR was obtained using a random effect meta-analysis. Triangles are proportional to the study size, (value of population size for each study is also given). Diamonds represent overall results. Horizontal lines represent 95% confidence intervals. Year, year of data collection. Id, article reference number. I^2^, statistic which measures the degree of heterogeneity. 95%CI, 95% confidence intervals. Id, article reference number. Weight (D+L), random effect weight based on the sample size of studies and degree of heterogeneity.

Overall, 7 studies provided data for alcohol abuse (fig. 6). The association was weak and not significant in Codecasa [51], due to the small number of immigrants included in the study (n = 38) and in the study by Odone et al [55], a relatively large study. Meta-analyses of these studies that span over 20 years yielded an OR of 0.10 (0.01; 0.83), as shown in Figure 6, which indicates that immigrants with TB were less likely to abuse alcohol than Italians. There was some evidence of heterogeneity (I^2^ 85.4, p<0.001; tau2 = 2.174), but after the exclusion of the study by Odone et al. the most recent study in our meta-analysis (1996–2006) [55], both I^2^ and tau2 reduced to 0. The funnel plot was asymmetric but there was no evidence of bias (Harbord: intercept: −2.346; p = 0.383).

## Discussion

Italy, like most western European countries showed a progressive reduction in the frequency of TB over the last century, while in the last twenty-five years, the trend has been almost stable, possibly jeopardizing the attainment of the goal of TB elimination that is recently under the focus of WHO [5]. The current epidemiological situation of tuberculosis in Italy is characterized by a low incidence in the general population, the concentration of most of the cases in some risk groups and in certain age groups, and the emergence of MDR-TB. The two most affected population groups are elderly in the Italian population the foreign people. The elderly population is at increased risk of reactivation of latent infections due to progressive deterioration of both general conditions and immune system, caused by the aging process itself. On the contrary, FBP which account for the increase of TB in age classes younger than 65 and for the great majority of MDR-TB resistant cases, are at increased risk of developing the disease either for the high incidence rates of TB in their countries of origin, or for the social fragility which may arise from the migration process itself. Also, differences in culture may impact significantly on TB prevention, diagnosis and treatment in FBP which unlike the general population is also at greater risk of having an infection with MDR-TB [53].

We analysed the scientific literature over a temporal frame of more than 30 years (1988–2013) with a total of 18 scientific papers, which were included in the meta-analysis. [37]–[41], [46], [51].

### IDU

The measures of association revealed that for intravenous drug use the meta-analysis gave no conclusive results, furthermore none of the studies included in the meta-analysis was significant per se. However, after one–stage meta analysis stratified by age group in articles for which individual data were available [37]–[41], we were able to observe that young immigrants were less likely to use injectable drugs than Italians. As shown in the international literature [57], [58], the use of injectable drugs is an important risk factor for the circulation and delay of treatment of TB in developed countries. The use of opiates may influence in different ways the human susceptibility to TB, either through their direct effect on the immune system and particularly on the cell-mediated immune response[59], [60], or because they are often linked to social features like tobacco use, homelessness, alcohol abuse and incarceration which are commonly associated with TB. Moreover, IDU is an effective driver of HIV-1 among the young individuals in Italy, and HIV-1 infection was, during the years of examined studies, and still is the most important reason for the excess of TB incidence among IDUs. This fact, may explain the greater proportion of IDU among younger Italians suffering from TB than FBP. These considerations are also consistent with the observation that use of illegal drugs intravenously, particularly heroin and morphine, is more diffuse among youngsters and adults living in Western countries than in their counterparts from non-drugs producing developing countries[61] [56] possibly because of their different cultural traditions and/or religious beliefs.

### MDR-TB

The European and Italian TB epidemiological picture of the recent years shows a progressive increase in the number of cases resistant to first-line drugs, particularly in Eastern Europe and in migrants from high MDR-TB burden countries to low endemic areas. While in Italy over the last decade the TB notification has been stable at approximately seven cases per 100,000 people annually, the proportion of TB cases notified in FBP increased to 46% of the total and, at the same time, the proportion of MDR-TB cases in FBP rose to the 83% [7]. Concurrently it has been observed a decrease in the proportion of African-born persons with TB (from 51% to 30%), whereas there was an increase of TB in immigrants from Eastern European countries (from 16% to 33%) [53]. Given this picture, it is not surprising that most of the studies included in our meta-analysis, which spans a 30 year period, showed no significant association between MDR-TB and FBP. Older studies reflect an out-dated epidemiological situation preceding the great migratory influx from east European countries which has substantially changed the epidemiological picture of TB in Italy, as testified by data about migration obtained from ISTAT [62] (Fig. 3b). On the contrary, more recent studies reflect better the current epidemiological picture [53] (Fattorini et al collected about 2671 cases in FBP between 2008 and 2010). An in-depth analysis of all available individual data by random effect multilevel logistic regression (one-stage approach meta-analysis) provided evidence of an association between MDR-TB and immigration status (the association was strong and statistically significant even after age adjustment). Furthermore, after classifying countries by their endemic level of MDR-TB into groups according to the WHO criteria [1], we found that immigrants from medium and high risk countries retained their higher risk of developing MDR-TB even after moving to a low endemic country.

### Alcohol abuse

Analysing the alcohol abuse factor we found that alcohol consumption was consistently associated with an increased risk of TB among Italians. This result is not unexpected since in some foreign countries, such as Islamic countries, the consumption of alcohol is forbidden for religious reason. Measuring the percentage of FBPs from east EU countries, where alcohol consumption is not hindered by religious habits, in articles that provided individual data [37]–[41] we found that migrants from east EU accounted for only a 10.5% of the total sampled population. FBP from east EU represented instead the 37% of the FBP in the paper by Odone [55] and excluding this study from the analysis, the overall OR remained pretty much the same because of the low number of FBP but the heterogeneity was reduced to zero (see results). Alcohol consumption at a level of 40g or more per day has been linked to an increased risk of TB although it is not clear if this is due to a direct effect of alcohol on the immune system or to specific social mixing patterns of alcohol abusers, which may increase the risk of exposure to people with infectious TB disease in settings such as bars, shelters for homeless, prisons, and social institutions [63]. Although among Italians alcohol abuse may be regarded as a marker of social vulnerability, and thereby constitute a risk factor for TB, the same cannot be said for the foreign population. Since official sources do not record alcohol consumptions for Italian and immigrants at the national level it is not possible to compare our findings with the general population.

### HIV status

A total of 10 articles were available for meta-analysis in relation to HIV status: grouping all the articles together we cannot detect a higher risk of developing TB associated with the HIV carrier state in FBP, however, stratifying the articles according to the introduction of cART for HIV it was observed that FBP had an increased risk of developing TB: HIV infection proved to be a time sensitive factor and the year 1996, when the cART became available, represented a cutting point. Since the effect of anti retroviral therapy is highly protective towards all the opportunistic infections usually associated with AIDS, TB included, it is obvious that a non-discriminatory access to this therapy is a crucial factor for the subsequent development of TB disease in latently infected individuals. Indeed, an unbalanced access to therapy of HIV infected individuals has been reported in many developed countries as a consequence of several socio-economic risk factors, such as race, income, intravenous drug use or illegal status [64]–[66]. In Italy, data from the AIDS Surveillance System show that FBP are associated with an increased risk of lack of any treatment before the AIDS diagnosis [67]. Furthermore, analysing the effect of age and length of stay in Italy on the likelihood to be HIV positive, we found that after spending 5 years in Italy, TB foreign-born patients have the same risk as Italians to be HIV positive, while the highest risk is found for subjects that have lived in Italy for less than 2 years. Overall, an increase in age was associated with an increased risk to be HIV infected; in the international literature also, both older age and recent arrival in a host country are factors commonly associated with an increased risk to develop TB [68], [69].

### Recurrent TB

Finally, when we took into consideration previous TB treatment as a proxy for recurrent TB, we observed the paradoxical result of FBP being less prone than Italians to recurrent TB. This result was quite consistent in the majority of the articles, Baussano[48] being the only exception because it presented a weak and not significant association between relapse and previous TB treatment. We repeated our analysis excluding this study and obtained almost the same value of association. The reason for the apparent discrepancy between most of the articles and Baussano resides in Baussano's distribution of the study population according to the country of birth: he assumed that the access to the national health system is the key issue to identify both new and previously treated TB cases and he put Italians and FBP from east Europe in the same category attributing them the same ability to access NHS; all the remaining cases went into the FBP non EU countries category. Such assumption however, may prove to be not completely true. Although highly consistent, we suggest that the association between previous treatment and Italians has to be considered with a certain caution because it may simply represent the inability of the public health system to follow up TB cases in FBP instead of a more resilience of immigrates to relapsing TB compared with Italians. Indeed, several epidemiological considerations point to the opposite: FBP are more likely than Italians not to be settled and consequently not to have a historical medical record that may report on previous TB treatment and more likely to stop, or not correctly fulfil the anti TB treatment [70].

In conclusion, our work on the factors possibly associated to TB in FBP gives back a complex picture that mostly depends upon the large time frame investigated and the changing epidemiological structure of the immigrant population during these 3 decades. According to the Italian National Institute of Statistic (Istat) data at 1 January 2013, there were 4,370,317 foreign residents accounting for 7.4% of the total population. About half of the foreign residents come from Eastern European countries: the Romanians, with nearly one million residents, represent the first foreign community; beside them, the more represented foreign communities are: Albanian, Moroccan, Chinese and Ukrainian. Migratory fluxes from the countries that have recently joined the EU have replaced those coming from the countries of North Africa that were very strong until the nineties[71].

We observed that a HIV positive status in a context of restricted access to combined anti-HIV therapy is more frequently associated to TB FBP after the introduction of c-ART in 1996, and that FBP are more frequently affected by multi drug resistant TB. Other factors depending on specific ethnic or cultural background or on a certain social fragility inherent to the immigrant status may also possibly be associated to TB-affected FBP. Finally, to our advice, the association of relapsing TB with Italian people is to be considered with extreme caution since it may well reflect the hidden universe of controversial approach of documented as well as non-documented FBP to public health in Italy.

Italy, like the majority of western European countries, is now home for millions of migrating people presenting with their specific burden of disease that includes latent TB status among others; therefore, the issue of an effective control of TB among migrants should be acknowledged as a cogent pre-requisite to reach the goal of TB elimination, in Italy as well as in other European countries since TB does not respect borders[72]. -

### Limitations of the study

We identified some limits of our-meta-analysis. First, some low quality papers, most of them describing studies carried out before 1996, were excluded from the meta-analysis because did not provide sufficient data on the factors under evaluation for either Italians or foreign-born TB patients. This may have introduced both selective bias (missing outcome, missing summary data) and small study effect bias in the meta-analysis of TB and HIV before 1996. Second, information bias may have distorted the results of individual studies if the misclassification of exposure was differential (e.g. difference in the amount and quality of information contained in the medical records of foreign born patients and Italians may have affected differentially the allocation of patients to risk categories) and this may be responsible for the high variability in the odds for alcohol. Another factor that may be responsible for the high degree of heterogeneity observed in the meta-analysis TB-alcohol is of course attributable to the changing racial and ethnic composition of immigrant populations entering Italy increasingly from Eastern Europe as opposed to Africa or Latin America, after the collapse of communism. In addition, immigrants were defined differently in different studies (e.g. foreign born patients, migrants, immigrants): for example Baussano[48] in his article have classified patients as born in the EU or elsewhere. This may have introduced bias in our meta-analysis if the EU born were more (or less) likely to gain access to the Italian national health system if their country of origin was Italy (or not Italy). Hence we carried out a sensitivity analysis excluding these studies. Third, confounding could limit the validity of our findings (e.g. low socio economic condition, age). To investigate whether differences in the distribution of confounding factors biased our results, we thoroughly investigated the sources of heterogeneity (e.g. age, length of stay, nation of birth, cART) and examined the association between the size of the effect and potential confounding when data on potential confounders where available (e.g. we were not able to rule out the effect of low socio-economic position in the association TB-alcohol intake, TB-HIV, TB-IDU and TB-relapses, because this information was not available in most studies). Forth, we performed stratified analyses to investigate whether the exposure-TB relationship was any different across levels of potential effect modifiers (e.g. immigrants were less likely than Italians to be ID users if aged less than 30 whereas the association was weak and not statistically significant for the older age-group). In the presence of effect modification (interaction), separate measures of effect for each level of the effect modifiers were presented.

## Supporting Information

Checklist S1 **PRISMA checklist.**
(DOC)
